# Using corpora to reveal style in translation: The case of *The Song of Everlasting Sorrow*

**DOI:** 10.3389/fpsyg.2022.1034912

**Published:** 2022-10-28

**Authors:** Lingzi Meng, Feng Pan

**Affiliations:** ^1^School of Foreign Languages, East China Normal University, Shanghai, China; ^2^School of Foreign Languages, Huazhong University of Science and Technology, Wuhan, Hubei, China

**Keywords:** corpus-based translation studies, translator’s style, Wang Anyi, *The Song of Everlasting Sorrow*, corpus keywords

## Abstract

This article reports on a corpus-based study of the English translation of Wang Anyi’s award-winning novel, *The Song of Everlasting Sorrow* [长恨歌, *Chang Hen Ge*] from the perspective of style. Using the keyword and concordance functions of corpus software packages AntConc and ParaConc, this research focuses on how the translator’s style reveals itself in the target text (TT) as well as how the style of the source text (ST) is represented in the TT. Findings show that the translators have a preference for contracted negative verb forms and expressions such as “bit” over “little,” making the text more colloquial. In general, the rendering of the ST style tends not to be entirely faithful. A distinction between reader-centered and text-centered keywords is made. While the reader-centered ST keywords are, as expected, largely altered, the translation of text-centered ST keywords is also rewritten, contrary to expectations. Presumably, the translators tended to reduce the ambiguity of the ST, resulting in a more explicit TT. The article argues that the translators chose to rewrite the translation to make it more understandable for the target audience since it concerned a work from a source culture very different from the target culture.

## Introduction

This study investigates how the style of a source text (ST) is represented in a target text (TT) as well as how the translator’s style reveals itself in the TT. The corpus of this research is the English translation of *The Song of Everlasting Sorrow* by Michael Berry and Susan Chan Egan of the Chinese novel 长恨歌 [Chang Hen Ge] by Wang Anyi. A mixed-method methodology is applied using corpus techniques (AntConc and ParaConc).

Style is one of the primary concerns of literary translation and can be defined as: “the linguistic characteristics of a particular text” (Leech and Short, 2007, p. 11). It is “the linguistic choices evident in a text [that] contribute to the overall meanings and effects of that text” (McIntyre and Walker, 2019, p. 16). The style of a translated text or the translation style, is supposed to be a mixture of the style of the original work and the translator’s style (Saldanha, 2011, p. 26). A translator’s style, known also as his/her “thumbprint” (Baker, 2000, p. 245), is “a matter of patterning: it involves describing preferred or recurring patterns of linguistic behavior, rather than individual or one-off instances of intervention” (Baker, 2000, p. 245); it is a “way of translating” that “distinguishes the translator’s work from that of others” (Saldanha, 2011, p. 30). Until now, corpus-based translation studies on style have focused on how the ST style is represented in the TT or on how the translator’s style reveals itself in the TT. How exactly this happens is still largely to be investigated. Most studies on translational style take an ST-oriented perspective, regarding the style of the TT as “a way of *responding* to the source text [style]” (Saldanha, 2011, p. 27, original emphasis) while not giving adequate consideration to the translator’s responsibility for the TT style (Bosseaux, 2004; Winters, 2007; Ji, 2009; Čermáková, 2015, 2018; Mastropierro and Mahlberg, 2017; Ruano, 2017; Malmkjær, 2018; Mastropierro, 2018a,^2020^; Toolan, 2018; Chen, 2019). Studies that are TT-oriented are usually concerned with the translator’s thumbprint and they fall into two sub-groups. One group, represented by Baker (2000), follows a TT-exclusive approach, leaving out the ST (Olohan, 2004; Saldanha, 2011; Mastropierro, 2018b). As noted by Saldanha (2011), the problem with this approach is that the style may be “carried over from the source text” (p. 32) and may not be a result of the translator’s mediation. Although this group of studies assumes that the translator’s style is an integral part of the TT style, they do not show how this style is related to the TT style. The other group adopts a parallel model where two or more TTs of the same ST are first compared between them and then with the ST (Winters, 2009; Li et al., 2011; Wang and Li, 2012; Meng, 2015). While parallel model studies effectively attribute specific stylistic choices to a particular translator, they ignore the ways in which TT style depends on the style of the translator. Moreover, they suffer from a methodological drawback: texts with more than one translated version in the same language are, in any case, rare.

Both ST-oriented and TT-oriented studies rely on specific pre-selected linguistic elements as indicators of style. For instance, Baker (2000) in her ground-breaking corpus-based study on translator’s style says about reporting verbs such as “say” that “[r]eporting structures in fiction and (auto)biography are both common and important […] for interpreting the role of the narrator and supporting characterization. We might expect a high level of variation here […]” (p. 251). In his corpus-based study of four Spanish translations of Dickens’s *Hard Times*, Ruano (2017) claims that the rendering of specific speech verbs is “fundamental if Dickens’s style and his individualization of the fictional voices is to be preserved” (p. 96). These assumptions can be challenged if we acknowledge that style can only be revealed in comparison, or in the words of Leech (2008), that “[t]o be stylistically distinctive, a feature of language must deviate from some norm of comparison” (p. 55).

This study investigates to what extent the ST style is transferred into the TT and how the translators reveal their “thumbprints” in the TT. The style markers of the ST and the TT, its keywords, are generated by comparing the texts with a “norm” rather than assuming them. Since the TT for the case study is currently the only English translation of the ST, a parallel-comparative model based on parallel and comparable corpora is adopted for this study, where the translators’ “thumbprint” is sought by comparing the translation with both a “norm” and the ST. This article contributes to the study of the translator’s style with a model that can detect the translator’s style when an ST has only one TT.

“Key-words provide a useful way to characterize a text or a genre.” (Scott, 2019, online). Using “keywords” to uncover the stylistic makeup of a literary work is common in corpus stylistics (McIntyre and Walker, 2019). However, the function of keywords in revealing translation style is under-explored. Kenny (2001) uses keywords to facilitate the discovery of creative language use in the translation. Čermáková (2015, 2018), from a practical perspective, discusses the importance of translators’ awareness of ST keywords in order to show a ‘particular consistency’ (2018, p. 123). To explore how ST cohesion is represented in the TT, Mastropierro and Mahlberg (2017) use keywords to help identify nodes that form cohesive networks in the ST that are then compared with the translation. In a few studies on the translator’s style, the TT keyword list is used to reduce the number of items for further study (Winters, 2009) or to help to determine meaningful word groups to look at as indicators of the translator’s style (Wang and Li, 2012). A different perspective is adopted by Johnson (2016). She considers both ST and TT keywords, and instead of comparing the texts with a comparable corpus, she makes an intra-text comparison, comparing the two parts of the same text. Her comparison between ST and TT concentrates on the keywords, while the translation contexts in which these keywords occur are left out. The next section introduces previous studies on *The Song of Everlasting Sorrow*, followed by a description of the methodology.

## Chang Hen Ge and *The Song of Everlasting Sorrow*

The novel *Chang Hen Ge*, winner of the Mao Dun National Prize for Fiction^1^ in 2000, was written in Mandarin Chinese by Chinese writer Wang Anyi [王安忆] (1954-) and published in 1995. It was translated into English by Michael Berry and Susan Chan Egan as *The Song of Everlasting Sorrow* (2008). In 2009, the translation received an honorable mention for the Sixth Lois Roth Award for the Translation of a Literary Work, and in 2011 it was nominated for the Man Booker Prize.

According to Wu (2012), Michael Berry spent 8 years working on his translation and visited Shanghai several times. In an interview, Berry stressed that he sought to be transparent and tried different English styles to get as close to the original as possible (Wu, 2014, p. 49). Although Berry and Egan admitted that the translation contained a series of stylistic deviations, they referred not so much to the language style as to the length of paragraphs and sentences, the adding of inverted commas to dialogues, and the italicization of a few words (Berry and Egan, 2008, p. vii).

Studies claim Berry and Egan’s translation is largely faithful. Zhu and Yang (2017), focusing on culture-specific items, suggest that the translators conveyed the exotic in the novel with slight loss, although they made minor adjustments to some expressions which seemed impossible to translate literally. Wu (2012) also noted the faithful representation of “otherness” in the translation, even when this went against Western literary aesthetics. These studies, while showing that the translators are skillful in rendering culture-loaded expressions, also testify to their high Chinese proficiency.

Some corpus-assisted studies, however, came to a different conclusion. Focusing on the application of degree adverbs, Wang and Li (2015) found that the translators tended to use English adverbs that emphasized the tragedy experienced by the heroine, enhancing the dramatic effect. At the textual level, Wang’s (2018) study analyzed how the introduction of paragraph breaks affected narratological features. Comparing the first two chapters of the ST with the TT, Wang found that the added paragraph breaks weakened the effect of the theme-relevant prolepses in the ST, even to the point of changing the plot.

Although these studies show style changes, they only consider a specific, pre-selected group of words or only examine a small part of the novel, thus leaving much space for further discussion. The present study revisits these conclusions from the perspective of a corpus-based analysis.

## Materials and methods

The keywords function of the AntConc generates a list of words by comparing a text with a reference corpus. The keyness feature shows the statistical significance of each occurrence. Usually, the list contains three types of keywords: proper nouns, indicators of the “aboutness” of a text, and style indicators (Scott, 2019, online). Jonathan Culpeper states that “[s]tyle markers as words whose frequencies differ significantly from their frequencies in a norm are precisely what keywords are” (Culpeper, 2009, p. 33). Keyword research should thus reveal “the most significant lexical differences between [texts], in terms of aboutness and style” (Baker, 2004, p. 237).

For this study, the ST and TT were paralleled at the sentence level using ParaConc (Barlow, 2009), a software program for parallel analysis, so that the ST concordances containing a given ST keyword could co-occur with the TT concordances and vice versa.

Two reference corpora were built to be compared with the ST and the TT, respectively. The Chinese reference corpus (CRC) which was used to reveal the ST textual style, consists of four novels, selected according to the following criteria: (i) winners of the Mao Dun National Prize for Fiction, and (ii) translated into English.^2^ The English-language reference corpus (ERC) applied to the exploration of the translation style (a result of the combination of the ST style and the translators’ style), was compiled from COCA sample data downloadable from https://www.corpusdata.org/formats.asp.^3^ While the sample contains texts of different genres, only works of fiction were fed into the ERC. The purpose of comparing the TT with a reference corpus justifies the selection of the fiction section of COCA as data for the ERC. Different from the aim of using the CRC to generate an ST keyword list that indicates the ST textual style, the application of the ERC was expected to generate a keyword list that contained both elements induced by the ST style transfer and those indicating the translators’ style. Since a translator’s style was “the translator’s characteristic use of language, his or her …preferred or recurring patterns of linguistic behaviour” (Baker, 2000, p. 245), it can be disclosed, at least to some extent, by comparing his or her translation with a reference corpus that represented the linguistic norm of the target language in the same genre as the translation. In other words, the reference corpus should be representative enough to reflect the norm and be made up of texts in the same genre as the TT and published in the same period. Designed with a balanced structure, COCA, “the only large and ‘representative’ corpus of American English”^4^, provides the ideal norm of language use against which the American-English TT can be compared. On the other hand, the sample data which were drawn randomly from fiction published from 1990 to 2012 was representative of the contemporary linguistic norms in the same broad genre of fiction as the TT, and thus a comparison between it and the TT can generate features that reveal the translators’ linguistic style. Table 1 shows the size of texts and corpora. In addition to the DIY corpora, the fiction section of COCA will be referenced in the discussion of the findings.

**TABLE 1:** Size of texts and corpora.

	ST	CRC	TT	ERC
Tokens (character or word)	266,921	979,029	196,461	361,938

To extract keyword lists for both ST and TT, the electronic version of each text was queried using AntConc 3.5.9 (Anthony, 2020), with a log-likelihood setting of *p* < 0.0001 and Hardie’s Log Ratio as the keyword effect size measure with 2 as the threshold. AntConc produced a list of 192 keywords for the ST and 84 for the TT (see Supplementary Appendices). The keyword list for the ST contained mainly double character words and multi-character and single-character words. Because the segmentation of the ST at the word level is not entirely accurate,^5^ a one-character word can be part of a word that was misrecognized. Single character words, 30 in total, were disregarded since they might not be bounded.

Both ST and TT keywords included adverbs, verbs, and adjectives, but primarily nouns. The nouns (and pronouns in the ST list), which are “words that human beings would recognize” (Scott, 2019, online) and reveal the “aboutness” of the novel. The other words show the style of the text. Nominal keywords are central to the faithful translation of the content (cf. Čermáková and Fárová, 2010) or when a literary text is analyzed from the perspective of literary criticism (cf. Mahlberg and McIntyre, 2011). In this study, nouns and pronouns among the keywords are not considered since they seem irrelevant to the style. This does not mean that style and aboutness are separable. Certain words have more to do with the style, while others have more to do with the aboutness of a text. The ST verbs 打针 [take an injection] and 打牌 [play cards] and the TT adjective *western* have also been left out of consideration, as they have more to do with the aboutness of the story than with the style. The TT keyword *Mr* has also been ignored because it is used exclusively as a title for two male characters in the ST: 程先生 (Mr. Cheng) and 严先生 (Mr. Yan). A problem is that the Chinese words 一些 [some], 有些 [some/a little], 一般 [as if/like] and 活跃 [lively] belong to more than one part-of-speech category. The word 一般, for example, can be an adverb or an adjective. These words are placed in the category in which they appear most frequently.

Previous studies on style and translation have paid little attention to static verbs as a style indicator. Wang and Li (2012) found that the translators of two Chinese versions of *Ulysses* showed a different style of adopting Chinese action verbs. Mastropierro (2020) and Ruano (2017) show how changes in the translation of reporting verbs affect characterization. Static verbs have yet to be investigated. In addition, function words indicating style have not been sufficiently studied, as some studies show that articles and prepositions are important style indicators (Culpeper, 2009; Wang and Li, 2012; Mastropierro, 2018a,b). Culpeper’s (2009) study suggests that subordinate adverbs such as “if” can explain characterization in *Romeo and Juliet*. Mastropierro (2018b) finds that conjunctive adverbs along with participles such as the cluster “and a,” can be markers of the translator’s idiosyncrasy. The present study considers how certain Chinese static verbs and function adverbs operate as style indicators and how their translations alter the style.

Different ways of categorizing keywords have been proposed to meet specific research goals (Culpeper, 2009; Mahlberg and McIntyre, 2011; Johnson, 2016). In their study of Ian Fleming’s *Casino Royale*, Mahlberg and McIntyre (2011) categorize keywords into “text-centered” and “reader-centered” (pp. 211–212). Although Mahlberg and McIntyre focus only on nouns, their categorization of text-centered and reader-centered keywords is useful and adopted here. Text-centered keywords imply low subjectivity, as they consist of “relevant links to characters, objects and places.” The reader-centered category “contains mainly abstract and metaphorical meanings that require more complex interpretation” and therefore “relates to the effects that the text creates on the reader at a higher level of abstraction” (Mahlberg and McIntyre, 2011, p. 211). The meaning and function of text-centered words are context-independent, while the meaning of reader-centered words is ambiguous and may change according to the context, leaving space for interpretation. The identification and classification of the keywords into either group are done independently by the two authors of the article in accordance with the criteria that whether the meaning of the ST keywords could lead to more than one interpretation with the concordance lines as context. When discrepancy occurred, a third analyst who also specializes in literary translation studies is consulted until an agreement is reached. Words whose meaning depended on the context are categorized as reader-centered, the others as text-centered. The hypothesis is that the translation of the text-centered keywords is faithful, while the ambiguity of the reader-centered keywords would be sacrificed to a certain extent.

Table 2 shows ST and TT keywords, with literal translations and part-of-speech indications for the ST words.

**TABLE 2:** Source text and target text keywords.

ST keywords		TT keywords
Text-centered	Reader-centered	
其实 adv. [actually]	有点 adv. [a little]	Didn’t
因此 adv. [therefore]	有些 adv. [a little/some]	Couldn’t
总之 adv. [in short]	做人 v. [conduct oneself/be a proper person]	Wasn’t
难免 adv. [hard to avoid]	一会儿 adv. [a while]	Wouldn’t
总是 adv. [always]	穿行 v. [go through]	Cannot
看上去 v. [look]	一般 adv. [as if/like]	Don’t
叫做 v. [be called]	觉着 v. [feel]	Isn’t
倘若 adv. [if]	有着 v. [have]	Doesn’t
有时 adv. [sometimes]	一些 adv. [some]	Weren’t
不由 adv. [cannot help]	好像 [as if/like]	Gradually
只得 adv. [have to]	带有 v. [have]	Simply
渐渐 adv. [gradually]	无所谓 v. [do not care]	However
不料 adv. [unexpectedly]	活跃 adj. [lively]	Neither
听见 v. [hear]	没什么 adv. [no/little/few]	Bit
晓得 v. [know]		Proper

## Findings and discussion

While elements of the ST style were transferred into the TT, the translators also left their fingerprints on the TT. The translation of reader-centered ST keywords was essentially unfaithful, but a significant proportion of the text-centered ST keywords were also rewritten. The translators eliminated ambiguousness, leaving less room for other interpretations. This resulted in alterations in characterization and narration. Tables 3, 4 show the translation strategies for the TT and ST keywords, excluding the TT verbs which are shown in Table 5 in the following sub-section.

**TABLE 3:** Translation strategies for the TT keywords.

	Occurrence	Keyness	Effect size	Mutation		Modification		Equivalence	
				*n*	%	*n*	%	*n*	%
Bit	161	118.33	2.1351	40	24.84	30	18.63	91	56.52
Gradually	65	96.02	4.0977	17	26.15	6	9.23	42	64.62
Simply	94	93.6	2.7369	72	76.6	0	0	22	23.4
However	117	90.25	2.2108	48	41.03	3	2.56	66	56.41
Neither	77	60.96	2.2046	2	2.6	2	2.6	76	94.8
Proper	42	47.22	2.987	15	35.71	8	19.05	19	45.24

**TABLE 4:** Translation strategies for the ST keywords.

	Occurrence	Keyness	Effect size	Mutation		Modification		Equivalence	
				*n*	%	*n*	%	*n*	%
有些 adv.	404	709.75	3.0767	249	61.9	73	18.91	83	21.5
一些 adv.	260	361.02	2.5672	84	32.31	95	36.54	81	31.15
好像 adv.	170	197.18	2.2557	54	31.76	16	9.41	100	58.82
有点 adv.	130	178.22	2.5438	102	78.46	14	10.77	14	10.77
觉着 v.	78	137.23	3.0886	31	39.74	17	21.79	30	38.46
有着 v.	67	136.19	3.4973	26	38.81	30	44.78	11	16.42
一会儿 adv.	113	115.44	2.067	29	25.66	36	31.86	48	42.48
一般 adv.	74	79.86	2.1459	32	43.24	7	9.46	35	47
活跃 adj.	31	71.98	3.9704	4	12.9	15	48.39	12	38.71
做人 v.	30	64.48	3.6821	14	47	11	36.67	5	17
没什么 adv.	40	62.17	2.7975	1	2.5	20	50	19	48
穿行 v.	18	60.22	6.6455	8	44.44	8	44.44	2	11.11
无所谓 v.	19	45.92	4.1386	4	21.05	8	42.11	7	36.84
带有 v.	21	39.72	3.283	5	24	15	71.43	1	4.76
其实 adv.	252	443.19	3.0866	165	65.48	3	1.19	84	33.33
倘若 adv.	62	186.19	5.4298	9	14.52	2	3.23	51	82.26
有时 adv.	83	146.44	3.0958	12	14.46	2	2.41	69	83.13
晓得 v.	49	133.99	4.7684	5	10.2	15	30.61	29	59.18
难免 adv.	49	110.1	3.8424	38	77.55	5	10.2	6	12.24
渐渐 adv.	79	101.24	2.4218	36	45.57	6	7.59	37	46.84
总是 adv.	101	98.59	2.0045	41	40.59	24	23.76	36	35.64
不由 adv.	67	92.85	2.5644	49	73.13	3	4.48	15	22.39
听见 v.	63	75.25	2.3032	15	23.81	6	9.52	42	66.67
看上去 v.	45	62.71	2.5752	16	35.56	4	8.89	25	55.56
不料 adv.	40	56.12	2.5881	14	35	12	30	14	35
因此 adv.	44	53.11	2.3203	28	63.63	3	6.82	13	29.55
只得 adv.	35	47.3	2.5174	28	80	1	2.86	6	17.14
叫做 v.	22	46.34	3.6131	6	27.27	3	13.64	13	59.09
总之 adv.	19	45.92	4.1386	13	68.42	0	0	6	31.58

**TABLE 5:** Occurrences of contracted and non-contracted forms of negation in the TT.

Contracted form	Keyness	Effect size	Occurrence	Non-contracted form	Occurrence
Didn’t	579.26	9.9964	277	Did not	171
Couldn’t	347.08	9.2577	166	Could not	78
Wasn’t	183.97	8.3421	88	Was not	123
Wouldn’t	81.53	7.1681	39	Would not	24
Don’t	258.98	6.938	133	Do not	18
Isn’t	52.26	6.5266	25	Is not	28
Doesn’t	43.9	6.275	21	Does not	23
Weren’t	39.72	6.1306	19	Were not	37

The labels “mutation” and “modification” are taken from van Leuven-Zwart (1989, p. 159). Based on her definition and criteria, in this study, mutation refers to adding, deleting, and using words that result in a complete meaning shift; modification refers to the strategies that result in a disjunction of meaning, without it being completely different. Cases of faithful translation are referred to as “equivalence.” Again, the grouping of the translation strategies is conducted manually by the two authors assisted by a third analyst when necessary. The following sub-sections elaborate on the findings.

### The translators’ style and the target text style

First, the translators preferred the contracted forms of negation, as evidenced by the use of *didn’t, couldn’t, wasn’t, wouldn’t, don’t, isn’t, doesn’t*, and *weren’t*. Since the Chinese do not have contractions, these forms do not come from the ST. Although non-contracted forms are also used in the TT, they are significantly less frequent or equally frequent, as shown in Table 5. The forms *wasn’t* and *weren’t* occur less often than the standard forms in the TT, but this does not affect the general trend. All these contracted forms show a high effect size (over 6) compared with the “norm.” Since the contracted forms of negation are less formal or emphasized than their counterpart two-word forms, the translators’ preference of the latter suggests that they tend to opt for a more casual style of narration.

As for the word *bit* that enjoys the highest keyness in the TT keyword list apart from the contracted forms (see Table 3), the analysis of the TT concordances shows that the translators used the word *bit* rather than *little* in adverbial phrases such as *bit by bit*, *a bit of*, and especially the pre-adjective mitigating adverbial phrase *a bit*. The phrases *a bit*, *bit by bit*, and *a bit of* occurring 128 times, 3 times, and 9 times, respectively in the TT, in contrast to the 31 occurrences of *a little* and the absence of *little by little* and *a little of*. This pattern is at odds with the occurrence of *bit* and *little* in the fiction section of COCA. In COCA fiction section, *a little* occurs 55,476 times, and *a bit* 15,950 times; the occurrence of *little by little*, 318 times, is about 1.5 times that of *bit by bit*, 193 times; the occurrence of *a little of* (567), although smaller than that of *a bit of* (3,832), also contrasts with its absence in the TT. This means that the translators prefer *bit*-constructions to *little*-contructions, although the latter is more widely used in American English. While *bit* is quite informal and mainly used in conversations, its adoption in the TT again indicates that translators tend to write in a casual style.

Regarding the TT style, Table 3 shows that most occurrences of *neither* (94.80%), *gradually* (74.62%), *bit* (56.52%), and *however* (56.41%) are equivalent to the ST expressions, indicating that the TT style follows the ST to some extent. Moreover, the Chinese equivalent for the word *gradually*, 渐渐, and that of the mitigating adverbial phrase *a bit* (occurring 128 times), 有些, also features in the ST keyword list, suggesting further that the TT style is partly the result of an ST style transfer. Regarding the adjective *proper*, the expression *proper young lady* is used 17 times (of the 19 times it appears as an equivalent of ST expressions) as an equivalent of the Chinese word 淑媛 [proper young lady], which also shows the ST influence on the TT style. However, the word *simply* in the TT keyword list is largely (76.60%) applied due to the translators’ mediation and reduces the interpretation space for TT readers. This tendency is discussed in the following.

### Representation of the source text style in the target text

#### Translation of reader-centered keywords

The ambiguity of the reader-centered ST words leaves readers plenty of room to develop their own interpretation. In the ST, degree adverbs such as 有点 [a little], 有些 [a little/some], 一些 [some], 一会儿 [a while], and 没什么 [no/little/few] express vague degrees or quantities; 觉着 [feel], 有着 [have], 带有 [have], as verbs of static or general meaning, are usually followed by abstract concepts that require active deciphering by the reader; 做人 [conduct oneself/be a proper person], 穿行 [go through], 无所谓 [do not care], and 活跃 [lively] are generally used figuratively; 好像 [as if/like] and 一般 [as if/like] imply uncertainty in speculation, judgment, or feelings. When these words occur, the narrator either sounds uncertain or gives only vague information. These words give the narrator and the characters, especially the male ones, a kind of feminine quality characterized by delicate perception and emotional mildness and restraint, reflecting the author’s intention to feminize Shanghai (Qi and Lin, 1995, p. 67; Nan, 1998, p. 69).

In the TT, the translators reduced or eliminated the original ambiguity by deleting or interpreting these words. As shown in Table 4, the equivalence rate for all reader-centered keywords, except 好像, is below 50%. This change affects narrative style and characterization. Examples^6^ 1–3 below, which show the translation of three adverbs, the keyness of which ranges from the highest to the lowest in the reader-centered keyword list (see the upper part of Table 4), illustrating the point.

Example 1

ST: 从片厂回来几天, 她都没什么表示, 这使吴佩珍沮丧。

[Several days passed after returning from the film studio, she gave few comments, this made Wu Peizhen depressed.]

TT: In the days following their visit to the film studio, Wang Qiyao did not utter a single word about their trip, and this left Wu Peizhen quite depressed.

Example 2

ST: 康明逊有些不安, 隐隐地有些明白, 几乎不敢再问, 可又不能不问。

[Kang Mingxun was a little disquieted, faintly had some guesses, almost dared not to ask any more, but thought he should ask.]

TT: Kang Mingxun was disquieted. He had some inkling of what she was up to; he was hesitant to ask but also felt he ought to press her for an explanation.

Example 3

ST: 可说到了这时, 王琦瑶才开始认真起来, 之前, 她就好像是应付蒋丽莉, 还应付程先生。

[It could be said that only till then did she begin to take it seriously, before then, she simply seemed to be dealing with Jiang Lili and Mr. Cheng perfunctorily.]

TT: Only then did she begin to take the pageant seriously; up until that point she had simply been trying to make Jiang Lili and Mr. Cheng happy.

The Chinese adverb of negation 没什么 [no/little/few] in Example 1 is important for both narrative style and characterization. Unlike 没/没有 [no], which always conveys a complete negation, 没什么 can express a complete or partial negation depending on the context. When the contextual information is not adequate or not available, different readers are likely to reach different conclusions. In Example 1, the narrator tells the readers about the heroine Wang Qiyao’s reaction after her visit to a film studio with her friend Wu Peizhen. The trip, arranged by Wu through her family connections, changes the course of the heroine’s life. The narrator tells the readers that although Wang is very interested in the studio, she does not tell her best friend Wu. So whether or not Wang makes any comments after their return is unclear. To make matters worse, the narrator is ambiguous about this, using 没什么表示 [gave few comments]. The heroine comes across as highly reserved to the point of being peculiar if we interpret the Chinese expression as meaning “there was no comment from her.” She would come across as less reserved and her behavior more palatable if the ST is taken to mean “a little comment from her.” In the TT, the translators opt for the first interpretation, which removes the ambiguity. Because of this explicitness, the TT narrator sounds authoritative while the heroine comes across as somewhat odd.

Example 2 describes the reaction of Kang Mingxun, Wang’s lover. She tells him she has come up with an idea to cover up that her illegitimate child is his. As mentioned earlier, all the male characters in the novel are slightly feminine. In this example, the word 有些 [a little], together with 隐隐地 [faintly], 几乎 [almost], and 不敢 [dare not], forms a descriptive cluster that constructs an emotionally restrained, sensitive, and mild image of a feminine man. The deletion of 有些 in the TT, among other changes, suggests that the character expresses his emotions more straightforwardly and seems more agitated than reserved. This deletion erases the vagueness and again helps construct a rather explicit narrator.

The ST in Example 3 tells the readers that the heroine changes her attitude after entering the second round of the beauty pageant. Before that, she deliberately appears indifferent toward the contest in front of Jiang and Cheng, who are making efforts to help her to do as good as possible in the beauty contest and uses this attitude as a protective shell for her dignity in case of failure. The word 好像 [seem] means that the narrator does not explicitly tell the readers. It is ambiguous whether the narrator knows the truth and refrains from telling it or whether she only views the character from the perspective of those around her. Either way, the ST readers cannot tell whether Wang really treats them indifferently or only pretends. The translation deletes 好像 [seem] and gives the TT readers only one version of the story, namely that Wang did not take the contest seriously. Also striking in this example is the addition of *simply* in the TT. It appears that the translators wanted to eliminate the indeterminacy and impose their own version. Example 4 provides a further illustration of the use of *simply*.

Example 4

ST: 是权力使然, 也是人生苦短。

[It was because of power, also because life was too short to do everything.]

TT: This was partly the result of what power does to a person, but it was also simply because he felt that life was too short to do otherwise.

The ST in Example 4 explains why Director Li, who later made the heroine one of his mistresses, is always chasing women. The readers can tell from the context that Director Li is a powerful official who uses women to relieve himself of the tensions and dangers he faces. The ST does not distinguish between the two reasons. However, the addition of *simply* in the TT puts extra emphasis on the second reason, while the added *partly* weakens the first one.

The verb 穿行 [go through] deserves attention because of its effect size, over 6.5, the largest of all the ST keywords, about 1.2 larger than the second largest. It is used nine times to describe inanimate objects, twice non-human creatures, four times general people and only twice to describe two of the main male characters, one being Kang, whose child the heroine has out of wedlock, the other being the man who kills her at the end of the story. This particular use of the verb suggests that even these two men, who determine the course of the heroine’s life, are like other nameless, faceless people or inanimate objects, implying the illusive nature of the heroine’s life. This is further emphasized by the fact that it is the only verb in the keyword list with a dynamic meaning. All other ST verb keywords (做人 [conduct oneself/be a proper person], 有着 [have], 带有 [have]) or verbs of sense or cognition (看上去 [look], 听见 [hear], 觉着 [feel], 无所谓 [do not care], 晓得 [know]) are static and do not describe observable actions. The whole fictional world seems to exist only in the minds of the characters and the narrator. Therefore, keeping this meaning of the verb in the TT may be necessary to stress the fictionality of the ST. However, the word was substituted in different contexts by English words of specific meaning, such as “crisscross,” “run,” “penetrate,” “move through,” and “shuttle back and forth.” As Table 4 shows, mutation and modification are the most common strategies used to translate this word, 44.44% each, which means that its potential meanings which result from its vagueness are lost in the translation.

#### Translation of text-centered keywords

Table 4 shows that high equivalence rate, over 55%, features only six text-centered keywords, which include four static verbs, 看上去 [look] (55.56%), 叫做 [be called] (59.09%), 听见 [hear] (66.67%) and 晓得 [know] (59.18%), and two adverbs, 倘若 [if] (82.26%), and 有时 [sometimes] (83.13%). This means that the hypothesis that the translation of the text-centered keywords is largely faithful does not hold.

The tendency of the translators to eliminate the ambiguity of the ST is extended to the text-centered keywords, particularly the adverbs. The group of adverbial text-centered ST keywords that are deleted or altered can be categorized into two sub-sets. One set, mainly occurring in the narration, includes adverbs that function as logical connectors and provide clues to the narrator’s covert attitudes or evaluations that encourage the readers to engage in a dialogue with her, which are 难免 [hard to avoid], 其实 [actually], 因此 [therefore], and 总之 [in short]. The word 难免, literally means *hard to avoid* and indicates a causal relationship between the result introduced by it and the cause described or implied in the context. It also suggests that the language user’s evaluation of the result is negative (Zhang, 2000, pp. 390–395). The word 其实 [actually] introduces a part of a sentence or a subordinate clause that complements or slightly contrasts the information given previously and implies that what follows is true, or truer, than what precedes. Instead of directly denying or simply deleting the previous information, the narrator gives more information that complements or contrasts it, leaving the readers with a more complicated picture. Unlike 难免 [hard to avoid] or 其实 [actually], neither 因此 [therefore] nor 总之 [in short] carry an evaluative meaning by themselves. In the ST, they function in a similar way as *in other words* or *similarly* which introduces another idea that elaborates on the previous one and acquires evaluative meaning when a comment that precedes them is repeated in what follows. As can be seen from Table 4, over two-thirds of occurrences of these Chinese adverbs, 难免 (87.75%), 其实 (66.67%), 因此 (70.45%), and 总之 (68.42%), are deleted, changed, or modified to give a version with an unambiguous meaning, leaving the TT narrator’s evaluation more explicit. Example 5 shows how 因此 (keyness 53.11), a neutral logic connector that is deleted or changed more often than the other neutral logic connector 总之 (keyness 45.92), is translated.

Example 5

ST: 这雨也不是什么倾盆的雨, 而是那黄梅天里的雨, 虽然不暴烈, 却是连空气都湿透的。因此, 这流言是不能小视的, 它有着细密绵软的形态, 很是纠缠的。

[The rain is not some torrent kind of rain, rather it is the kind of rain during the season when the plums are ripe, which though not violent, drenches even the air. Therefore, rumors are not to be underestimated; they have a fine, closely woven and soft form, entwining with you very tightly.]

TT: The rain comes down not in a torrent but as a hazy springtime drizzle. Although not violent, it drenches the air with an inescapable humidity. Never underestimate these rumors: soft and fine as these raindrops may be, you will never struggle free of them.

因此 [therefore] in Example 5, which connects the two sentences and functions in a comparable way as *similarly*, creates ambiguity in the narrator’s discussion of rumors. Before these sentences in the ST, the narrator compares rumors to clouds bringing rain that symbolizes problems and disputes. The image of 黄梅天里的雨 [rain during the season when the plums are ripe] is used to figuratively discuss the effect of rumors. The word 因此 suggests that these are almost the same as 黄梅天里的雨. The characteristics of rumors, 细密绵软的形态 [a fine, closely woven and soft form] and 很是纠缠的 [entwining with you very closely], depicted in the second sentence, are very much the characteristics of 黄梅天里的雨, which ST readers can recognize. However, they are deferred to describe rumors, rather than the rain in the first sentence. The narrator creates a kind of ambiguity that confuses rain and rumors. Another ambiguity is the contradiction caused by 因此. Although rumors are previously compared to the clouds that produce rain, in this sentence, 因此 suggests that these rumors are the rain and thus the troubles and disputes rather than their cause. In the TT, the implicitness and ambiguity in the comparison are eliminated, for 因此 is deleted and replaced by the direct comparison between rumors and rain, “soft and fine as these raindrops may be.” TT readers are given a version with an unambiguous meaning and are saved from the reasoning task set in motion by 因此.

The other set, the adverbs 只得 [have to], 不由 [cannot help], and 不料 [unexpectedly], are almost exclusively used to describe character actions, indicating that the characters are often driven by forces beyond their control. These words are also significantly rewritten: only 17.14, 22.39, and 35% of them respectively have an equivalent in the TT (see Table 5). With these words deleted or changed, the characters appear to have more control over themselves, which means that the original meaning potential that calls for readers’ active decoding is sacrificed. Example 6 illustrates this point with the translation of 不由, the word of the highest keyness (92.85) among the three adverbs.

Example 6

ST: 康明逊这才听出这一句句原来都是冲着他来的, 不由后退了几步, 嘴里嗫嚅着。

[Kang Mingxun only then realized that all these words were targeted at him, he could not help but make several steps backward, speaking haltingly.]

TT: Suddenly Kang Mingxun realized that he was the object of her wrath. He stepped back and stammered something inaudible.

In Example 6, 不由 [could not help] suggests that moving backward is not a deliberate action, but happens unconsciously, after realizing that he himself is the target of lashing and criticism in the form of innuendoes from Wang’s mother. She understood that Kang is the father of the child, and is outraged that he does not take responsibility. Although the narrator does not mention his inner feelings, the word 不由 suggests his complex emotions (surprise, shame, panic, and helplessness) that are not under his control, leaving room for the reader’s own interpretation. The TT, on the other hand, does not imply this, and his action of stepping back looks very conscious.

It is worth noting that the translation of the frequency adverb 有时 [sometimes] contrasts with that of 总是 [always], the other frequency adverb in the ST keyword list, of which about 65% is either deleted or modified, as shown in Table 5. The different translation strategies for the two adverbs also suggest that the translators wanted to avoid the original ambiguity in the TT. While the English equivalent of 有时, *sometimes*, carries no evaluative meaning, the counterpart of 总是, *always*, has a unique usage that both *sometimes* and 总是 lack. In some contexts, it does not literally mean that a particular action always happens but means that the action happens often. This hyperbolic use implies that the action or event is questionable or undesirable. Some occurrences of 总是 in the ST, if translated literally as *always*, could cause indeterminacy of meaning because it sounds as if the character or narrator were speaking hyperbolically. In this case, the translators chose other expressions or deleted the word to avoid ambiguity. It was kept if there was no possibility that the translation would be understood as a complaint about an undesirable event.

### Discussion

In the above sections, we have shown that translators tend to rewrite both the reader-centered and text-centered keywords of the ST. While these words, relevant to the overall feminine style of the ST, are ambiguous in meaning or function, the rewriting eliminates the vagueness and renders the TT rather explicit, thus altering the original style. It is curious that the translators, who have suggested that they are sensitive and attentive to the style of the works they translate (Wu, 2014, p. 49), make these stylistic changes in their translation. While their Chinese language proficiency has already been verified by previous studies (see section “Chang Hen Ge [长恨歌] and *The Song of Everlasting Sorrow*”), there is little possibility that the translators were not able to decipher the polysemy of the above-mentioned Chinese words. Most likely this was for the sake of readability. In the “translators’ notes,” the two translators said that “[f]or the purpose of readability in English” and to save the challenge to the reader (Berry and Egan, 2008, p. vii), their translation shows some stylistic variance from the original. Although by the style they only mean the length of paragraphs and sentences and the presentation of discourse, their translation principle of achieving readability in English must be applied to every aspect of the text, including translating the ST words of ambiguity in meaning and those requiring readers’ considerable interpretive effort. While meaning indeterminacy in the ST gives way to the readers’ active involvement, the translators as the first readers from the target culture produced a version based on their own understanding. On the other hand, since deciphering the ambiguities in the ST requires background knowledge that is likely to be highly culture-specific, a literal translation of ST ambiguous words could fail to open up the same multiple readings from TT readers as those from ST readers. This study argues that despite the fact that in most cases, translators are supposed to convey the original style to the target audience, they sometimes have to make a compromise when translating a literary work in a culture very different from the original culture.

Another reason for the stylistic unfaithfulness might be that the translators did not notice the stylistically significant-but-plain-looking words in their analysis of the ST before translating. While formal stylistic features such as those mentioned by the translators are usually easily identified, words that are important for style are likely to be subtle and dispersed throughout the text and therefore may be overlooked with the naked eyes. As shown by Čermáková and Fárová (2010), translators may not always be aware of the key status of some of the words in an ST, therefore missing some of the original meaning or altering the characterization. From a practical perspective, the present study implies that literary translators could be effectively aided by corpus in terms of the analysis of ST style before translating. Corpus techniques like KEYWORD can help reveal ST words or word groups that appear “plain” but are statistically and stylistically significant, disclosing hidden signposts for the translator’s textual endeavor.

This study also shows that both verbs and adverbs can be important indicators of style. Even function words, such as conjunctive adverbs, can take on an evaluative meaning that is important for the narrative style, while certain recurring dynamic verbs, represented by 穿行, are important for representing the deeper literary meaning of the fictional work. As a result, altering the meaning of these words in translation leads to stylistic changes that affect narrative style and characterization. It is also found that the translators used different translation strategies for the static and dynamic verbs. As shown in Table 4, the translators translated the text-centered verbs, which are all static, fairly faithfully, as the equivalent percentage of these verbs is above 55%. This pattern contrasts with the translation of the dynamic verb 穿行, and also differs from both Mastropierro’s (2020) and Ruano’s (2017) findings. In their respective studies on the translation of reporting verbs, they both found that the translations showed a large lexical variety and did not fully preserve the value of the verbs. These studies and the present study took into consideration together, it seems to suggest that literary translators tend to rewrite dynamic verbs to a greater extent than static verbs in their translations. But large scale studies across languages are needed to verify this preliminary point.

The parallel-comparative model proposed in this study proves effective for revealing the translator’s style to some extent. The translators are found to prefer the contracted forms of negation to their two-word counterparts for most auxiliaries and they usually opt for *bit* rather than *little* in constructions such as *a bit*, *bit by bit*, and *a bit of*, endowing the TT with a casual style from time to time. In other words, the model turns out to be useful in filtering out the influence of the ST, which the TT-oriented model would fail, and in singling out some of the translators’ characteristic use of language that distinguishes them from other translators when there is only one TT, in which case the ST-oriented model would not be workable. Nevertheless, it should be noted that these features do not account for a more generalizable description of the translators’ style. They only indicate some aspects of it. This implies that keywords alone cannot bring out the full capacity of this model, and other corpus techniques, such as clusters, should be applied to complement the search for the likely candidates for the translators’ style. Mastropierro’s (2018b) study has shown that key clusters, instead of keywords, can indicate stylistic differences between two TTs of the same ST. The parallel-comparative model can be fruitfully tested with CLUSTERS and other corpus techniques to reach a more integrated representation of the translators’ style, which calls for further research in the future.

## Conclusion

This study uses corpus techniques, particularly the KEYWORDS, to examine what are the features of the translators’ style and the transfer of the ST style represented in the English translation of *Chang Hen Ge*, *The Song of Everlasting Sorrow*. The findings are that the translators preferred the contracted negative forms of verbs and the word *bit* to *little*, suggesting that they write in a rather casual style. As for the translational style, the representation of the ST style in the TT tends not to be faithful. As expected, the reader-centered ST keywords were heavily rewritten. Contrary to expectations, the translation of text-centered ST keywords was also unfaithful to a considerable extent. It appears that the translators wanted to reduce the ambiguity, making the TT more explicit. The effects of the rewriting are that the TT readers are left with a more authoritative narrator who gives less room for their interpretation and that the characters in the TT are more in control of themselves, and less susceptible to outside forces. The rewriting thus fails to some degree to represent the author’s intention of feminizing Shanghai in the story. Our findings challenge claims voiced in other studies of the novel according to which the translation faithfully reproduces the original, but are consistent with the conclusions made by Wang and Li (2015) and Wang (2018) who state that stylistic and narratological differences exist between the ST and the TT. We argue that stylistic alteration is inevitable for the TT to achieve readability among readers who come from a culture very different from the one which the ST author and readers are from.

Despite the conclusion reached, it is essential to point out that the findings concerning the translators’ style are only preliminary. Even though some features of the translators’ style have been successfully revealed with the corpus KEYWORDS function, these features only represent part of the spectrum of indicators of the translators’ style. Future studies are expected to take into account a wider range of textual features (especially those above word level) that may indicate the translators’ style. Another point to be noted is that the register of the texts in the ERC, which covers both popular and serious literature, is not exactly the same as that of the TT, a novel of a serious theme. While we intended to use this reference corpus to reveal the general linguistic norm of the target language in the broad genre of fiction (as justified in section “Materials and methods”), future studies can build a reference corpus with texts of more comparable literariness to that of the TT to see if the results differ. We hope this preliminary research may constitute the first step toward more corpus-based studies on the translators’ style using the parallel-comparative model in the future.

## Data availability statement

The original contributions presented in this study are included in the article/Supplementary material, further inquiries can be directed to the corresponding author.

## Author contributions

LM contributed in the literature review, data analysis, and references of the study. FP designed the project and the methodology. Both authors contributed to the “Discussion” section and approved the submitted version.

## Footnotes

1 Named after renowned Chinese writer Mao Dun, the prize was created in 1981 to honor the excellent Chinese novels every 4 years and remains the most prestigious award for literature in the China mainland today.

2 The four novels are 穆斯林的葬礼 [*Mu Si Lin De Zang Li*], *Jade King* by 霍达 [Huo Da]; 都市风流 [*Du Shi Feng Liu*], *Metropolis* by 孙力 and 余小慧 [Sun Li and Yu Xiao-hui]; 少年天子 [*Shao Nian Tian Zi*], *Son of Heaven* by 凌力 [Ling Li]; and 兄弟 [*Xiong Di*], *Brothers* by 余华 [Yu Hua].

3 As noted on the website, the sample data are taken from COCA entirely at random from texts published from 1990 through 2012 (about 1/100th the total number of texts). The sample data are not cleaned up; consequently, there is some noise in them, such as messy codes @ and #. Limitations that may result from this should be acknowledged here. For one, the number of messy codes, though not large, impacts the statistics. Different types of fiction vary in terms of linguistic style, which might also influence the statistics of the keywords.

4 https://www.english-corpora.org/coca/, accessed 23 September 2022.

5 Words in Chinese text are written together without spaces between them. Therefore, Chinese texts are usually segmented (i.e., spaces are inserted between words or characters) with computer software for a corpus. Unfortunately, to date, no software achieves 100% accuracy in segmenting Chinese, including the software ICTCLAS used for segmenting the ST in this study.

6 All translations in square brackets are by the authors.
