# Supplementary material for: Using corpora to reveal style in translation: The case of The Song of Everlasting Sorrow
Source: Front Psychol. 2022 Oct 28;13:1034912. doi: 10.3389/fpsyg.2022.1034912 (PMC9650525; doi:10.3389/fpsyg.2022.1034912)
Supplement: Supplementary file 1 [file Table_1.DOCX]

Appendix 1. ST keywords sorted by keyness

|  | Occurrence | Keyness | Effect size | Keyword |
| --- | --- | --- | --- | --- |
| 1 | 2070 | 7810.53 | 14.491 | 王琦瑶 |
| 2 | 529 | 1991.85 | 12.5227 | 程先生 |
| 3 | 470 | 1769.55 | 12.3521 | 蒋丽莉 |
| 4 | 296 | 1114.18 | 11.6851 | 薇薇 |
| 5 | 225 | 846.84 | 11.2894 | 张永红 |
| 6 | 208 | 782.84 | 11.1761 | 萨沙 |
| 7 | 405 | 709.75 | 3.0767 | 有些 |
| 8 | 580 | 671.26 | 2.2504 | 便 |
| 9 | 176 | 662.38 | 10.935 | 李主任 |
| 10 | 167 | 616.59 | 9.8593 | 长脚 |
| 11 | 151 | 568.27 | 10.714 | 康明逊 |
| 12 | 147 | 553.21 | 10.6753 | 阿二 |
| 13 | 144 | 530.31 | 9.6455 | 弄堂 |
| 14 | 369 | 495.26 | 2.5032 | 些 |
| 15 | 353 | 468.75 | 2.4838 | 她们 |
| 16 | 123 | 462.88 | 10.4181 | 毛毛娘舅 |
| 17 | 124 | 455.32 | 9.4298 | 小林 |
| 18 | 252 | 443.19 | 3.0866 | 其实 |
| 19 | 116 | 436.53 | 10.3336 | 严师母 |
| 20 | 98 | 368.78 | 10.0903 | 严家师母 |
| 21 | 260 | 361.02 | 2.5672 | 一些 |
| 22 | 93 | 349.97 | 10.0148 | 吴佩珍 |
| 23 | 93 | 349.97 | 10.0148 | 老克腊 |
| 24 | 89 | 334.91 | 9.9514 | 邬桥 |
| 25 | 166 | 331.47 | 3.4413 | 它们 |
| 26 | 312 | 308.91 | 2.0229 | 它 |
| 27 | 72 | 270.93 | 9.6455 | 平安里 |
| 28 | 198 | 261.63 | 2.4756 | 上海 |
| 29 | 95 | 245.78 | 4.4605 | 一日 |
| 30 | 158 | 237.3 | 2.7241 | 房间 |
| 31 | 62 | 233.3 | 9.4298 | 爱丽丝 |
| 32 | 107 | 218.85 | 3.5166 | 马路 |
| 33 | 137 | 217.7 | 2.8457 | 虽 |
| 34 | 168 | 214.72 | 2.4167 | 城市 |
| 35 | 58 | 201.37 | 7.3336 | 流言 |
| 36 | 161 | 197.55 | 2.3471 | 样子 |
| 37 | 170 | 197.18 | 2.2557 | 好像 |
| 38 | 51 | 191.91 | 9.148 | 公寓 |
| 39 | 144 | 190.25 | 2.4756 | 则 |
| 40 | 62 | 186.19 | 5.4298 | 倘若 |
| 41 | 51 | 182.35 | 8.148 | 晚会 |
| 42 | 48 | 180.62 | 9.0606 | 时尚 |
| 43 | 130 | 178.22 | 2.5438 | 有点 |
| 44 | 123 | 173.48 | 2.598 | 因 |
| 45 | 43 | 161.8 | 8.9019 | 片厂 |
| 46 | 76 | 153.42 | 3.4756 | 暗 |
| 47 | 57 | 151.59 | 4.6081 | 窗帘 |
| 48 | 83 | 146.44 | 3.0958 | 有时 |
| 49 | 57 | 143 | 4.3085 | 导演 |
| 50 | 38 | 142.99 | 8.7235 | 上海小姐 |
| 51 | 92 | 141.21 | 2.7704 | 底 |
| 52 | 57 | 140.34 | 4.221 | 楼梯 |
| 53 | 77 | 139.12 | 3.1574 | 静 |
| 54 | 78 | 137.23 | 3.0886 | 觉着 |
| 55 | 67 | 136.19 | 3.4973 | 有着 |
| 56 | 49 | 133.99 | 4.7684 | 晓得 |
| 57 | 75 | 127.54 | 3.004 | 夜晚 |
| 58 | 44 | 127.08 | 5.1277 | 外婆 |
| 59 | 102 | 123.7 | 2.3279 | 旧 |
| 60 | 34 | 119.18 | 7.5631 | 摩登 |
| 61 | 31 | 116.65 | 8.4298 | 电车 |
| 62 | 113 | 115.44 | 2.067 | 一会儿 |
| 63 | 39 | 113.39 | 5.1761 | 旗袍 |
| 64 | 79 | 110.93 | 2.5896 | 一半 |
| 65 | 49 | 110.1 | 3.8424 | 难免 |
| 66 | 31 | 108.08 | 7.4298 | 闺阁 |
| 67 | 33 | 104.51 | 5.935 | 照相 |
| 68 | 73 | 103.02 | 2.5994 | 心情 |
| 69 | 31 | 102.22 | 6.4298 | 表哥 |
| 70 | 79 | 101.24 | 2.4218 | 渐渐 |
| 71 | 86 | 100.79 | 2.2725 | 灯 |
| 72 | 101 | 98.59 | 2.0045 | 总是 |
| 73 | 59 | 97.34 | 2.932 | 快乐 |
| 74 | 38 | 97.17 | 4.4016 | 地板 |
| 75 | 25 | 94.07 | 8.1195 | 娘姨 |
| 76 | 67 | 92.85 | 2.5644 | 不由 |
| 77 | 24 | 90.31 | 8.0606 | 二妈 |
| 78 | 26 | 89.61 | 7.1761 | 橱窗 |
| 79 | 28 | 86.64 | 5.698 | 苏联 |
| 80 | 39 | 85.71 | 3.761 | 且 |
| 81 | 22 | 82.78 | 7.935 | 聪敏 |
| 82 | 59 | 82.3 | 2.5769 | 电影 |
| 83 | 24 | 82.24 | 7.0606 | 竞选 |
| 84 | 74 | 79.86 | 2.1459 | 一般 |
| 85 | 34 | 79.1 | 3.9781 | 气味 |
| 86 | 21 | 79.02 | 7.8679 | 康乃馨 |
| 87 | 21 | 79.02 | 7.8679 | 鸽群 |
| 88 | 60 | 78.21 | 2.4518 | 灯光 |
| 89 | 28 | 75.65 | 4.698 | 后门 |
| 90 | 20 | 75.25 | 7.7975 | 严先生 |
| 91 | 20 | 75.25 | 7.7975 | 弄口 |
| 92 | 63 | 75.25 | 2.3032 | 听见 |
| 93 | 39 | 72.58 | 3.2375 | 类 |
| 94 | 34 | 72.53 | 3.6562 | 时光 |
| 95 | 24 | 72.46 | 5.4756 | 电梯 |
| 96 | 31 | 71.98 | 3.9704 | 活跃 |
| 97 | 19 | 71.49 | 7.7235 | 淮海路 |
| 98 | 19 | 71.49 | 7.7235 | 照相间 |
| 99 | 21 | 71.21 | 6.8679 | 打牌 |
| 100 | 62 | 71.18 | 2.24 | 窗 |
| 101 | 34 | 70.54 | 3.5631 | 男朋友 |
| 102 | 29 | 70.49 | 4.1637 | 打针 |
| 103 | 23 | 68.94 | 5.4142 | 舞会 |
| 104 | 45 | 68.67 | 2.758 | 镜子 |
| 105 | 24 | 68.66 | 5.0606 | 风情 |
| 106 | 18 | 67.73 | 7.6455 | 派推 |
| 107 | 32 | 66.5 | 3.5687 | 寂寞 |
| 108 | 44 | 66.11 | 2.7256 | 窗户 |
| 109 | 43 | 66.06 | 2.7726 | 灰 |
| 110 | 25 | 65.59 | 4.5345 | 面目 |
| 111 | 30 | 64.48 | 3.6821 | 做人 |
| 112 | 17 | 63.97 | 7.5631 | 婚服 |
| 113 | 17 | 63.97 | 7.5631 | 张妈 |
| 114 | 45 | 62.71 | 2.5752 | 看上去 |
| 115 | 40 | 62.17 | 2.7975 | 没什么 |
| 116 | 22 | 61.78 | 4.935 | 出场 |
| 117 | 18 | 60.22 | 6.6455 | 明星 |
| 118 | 18 | 60.22 | 6.6455 | 穿行 |
| 119 | 16 | 60.2 | 7.4756 | 后弄 |
| 120 | 16 | 60.2 | 7.4756 | 大小姐 |
| 121 | 25 | 60.16 | 4.1195 | 苏州 |
| 122 | 15 | 56.44 | 7.3825 | 夹竹桃 |
| 123 | 15 | 56.44 | 7.3825 | 开麦拉 |
| 124 | 15 | 56.44 | 7.3825 | 灶间 |
| 125 | 15 | 56.44 | 7.3825 | 酒精灯 |
| 126 | 40 | 56.12 | 2.5881 | 不料 |
| 127 | 40 | 56.12 | 2.5881 | 屋顶 |
| 128 | 27 | 55.45 | 3.5301 | 鸽子 |
| 129 | 20 | 54.95 | 4.7975 | 潮流 |
| 130 | 36 | 54.43 | 2.7387 | 忽 |
| 131 | 53 | 54.42 | 2.0743 | 太阳 |
| 132 | 47 | 54.26 | 2.2488 | 情景 |
| 133 | 31 | 53.44 | 3.0375 | 终 |
| 134 | 52 | 53.13 | 2.0675 | 道理 |
| 135 | 44 | 53.11 | 2.3203 | 因此 |
| 136 | 22 | 53.04 | 4.1277 | 情形 |
| 137 | 28 | 52.96 | 3.283 | 圣诞 |
| 138 | 14 | 52.68 | 7.283 | 外乡人 |
| 139 | 14 | 52.68 | 7.283 | 罗曼蒂克 |
| 140 | 39 | 52.58 | 2.5131 | 缝 |
| 141 | 46 | 52.06 | 2.2178 | 年纪 |
| 142 | 18 | 51.5 | 5.0606 | 歌舞 |
| 143 | 51 | 51.07 | 2.0395 | 反 |
| 144 | 34 | 50.58 | 2.7051 | 盏 |
| 145 | 46 | 50.32 | 2.1663 | 空气 |
| 146 | 27 | 50.12 | 3.2305 | 麻将 |
| 147 | 36 | 49.72 | 2.5581 | 自由 |
| 148 | 20 | 49.15 | 4.2126 | 镜头 |
| 149 | 13 | 48.91 | 7.1761 | 梦魇 |
| 150 | 13 | 48.91 | 7.1761 | 窗幔 |
| 151 | 40 | 48.8 | 2.3381 | 影 |
| 152 | 40 | 47.85 | 2.3057 | 玩 |
| 153 | 25 | 47.78 | 3.3121 | 遮 |
| 154 | 35 | 47.3 | 2.5174 | 只得 |
| 155 | 22 | 46.34 | 3.6131 | 叫做 |
| 156 | 19 | 45.92 | 4.1386 | 总之 |
| 157 | 19 | 45.92 | 4.1386 | 无所谓 |
| 158 | 12 | 45.15 | 7.0606 | 上海生活 |
| 159 | 12 | 45.15 | 7.0606 | 梅兰芳 |
| 160 | 12 | 45.15 | 7.0606 | 水道 |
| 161 | 12 | 45.15 | 7.0606 | 老张 |
| 162 | 16 | 44.62 | 4.8907 | 大妈 |
| 163 | 16 | 44.62 | 4.8907 | 钢琴 |
| 164 | 36 | 43.54 | 2.3236 | 说是 |
| 165 | 19 | 43.52 | 3.9162 | 年头 |
| 166 | 31 | 43.12 | 2.5718 | 颜色 |
| 167 | 38 | 42.51 | 2.2 | 时代 |
| 168 | 20 | 42.31 | 3.6276 | 咖啡 |
| 169 | 13 | 42.04 | 6.1761 | 寂寥 |
| 170 | 17 | 42.04 | 4.2412 | 面包 |
| 171 | 21 | 41.47 | 3.4085 | 前边 |
| 172 | 21 | 41.47 | 3.4085 | 景 |
| 173 | 11 | 41.39 | 6.935 | 下午茶 |
| 174 | 11 | 41.39 | 6.935 | 京剧 |
| 175 | 11 | 41.39 | 6.935 | 化妆间 |
| 176 | 11 | 41.39 | 6.935 | 晒台 |
| 177 | 11 | 41.39 | 6.935 | 暗房 |
| 178 | 11 | 41.39 | 6.935 | 桥牌 |
| 179 | 11 | 41.39 | 6.935 | 王小姐 |
| 180 | 19 | 41.32 | 3.7235 | 壳 |
| 181 | 14 | 41.28 | 5.283 | 光影 |
| 182 | 14 | 41.28 | 5.283 | 陡 |
| 183 | 32 | 41.23 | 2.4312 | 化 |
| 184 | 22 | 40.9 | 3.2346 | 照相馆 |
| 185 | 37 | 40.35 | 2.1615 | 痛 |
| 186 | 31 | 39.9 | 2.4298 | 墙上 |
| 187 | 31 | 39.9 | 2.4298 | 彼此 |
| 188 | 21 | 39.72 | 3.283 | 带有 |
| 189 | 30 | 39.62 | 2.4756 | 聚 |
| 190 | 17 | 39.55 | 3.9781 | 约会 |
| 191 | 19 | 39.3 | 3.5536 | 艳 |
| 192 | 16 | 38.8 | 4.1537 | 老妈子 |

Appendix 2. TT keywords sorted by keyness

|  | Occurrence | Keyness | Effect size | Keyword |
| --- | --- | --- | --- | --- |
| 1 | 1962 | 4113.79 | 12.8208 | wang |
| 2 | 1898 | 3979.2 | 12.773 | qiyao |
| 3 | 501 | 1048.06 | 10.8514 | jiang |
| 4 | 481 | 1006.19 | 10.7926 | lili |
| 5 | 460 | 962.23 | 10.7282 | cheng |
| 6 | 312 | 652.49 | 10.1681 | weiwei |
| 7 | 302 | 631.57 | 10.1211 | shanghai |
| 8 | 277 | 579.26 | 9.9964 | didn’t |
| 9 | 258 | 539.52 | 9.8939 | zhang |
| 10 | 226 | 472.58 | 9.7029 | yan |
| 11 | 222 | 464.21 | 9.6771 | yonghong |
| 12 | 182 | 380.54 | 9.3905 | wu |
| 13 | 178 | 372.18 | 9.3584 | sasha |
| 14 | 166 | 347.08 | 9.2577 | couldn’t |
| 15 | 469 | 337.95 | 2.1123 | mr |
| 16 | 182 | 332.6 | 5.8055 | director |
| 17 | 208 | 312.75 | 4.1908 | madame |
| 18 | 148 | 309.44 | 9.0922 | longtang |
| 19 | 137 | 286.43 | 8.9807 | xiao |
| 20 | 135 | 282.25 | 8.9595 | lin |
| 21 | 134 | 280.16 | 8.9488 | kang |
| 22 | 134 | 280.16 | 8.9488 | mingxun |
| 23 | 128 | 267.61 | 8.8827 | li |
| 24 | 133 | 258.98 | 6.938 | don’t |
| 25 | 124 | 240.44 | 6.8369 | colour |
| 26 | 115 | 240.43 | 8.7282 | peizhen |
| 27 | 106 | 221.61 | 8.6106 | maomao |
| 28 | 88 | 183.97 | 8.3421 | wasn’t |
| 29 | 244 | 182.1 | 2.1696 | heart |
| 30 | 96 | 160.26 | 4.8827 | bridge |
| 31 | 220 | 156.6 | 2.0942 | city |
| 32 | 73 | 152.61 | 8.0725 | deuce |
| 33 | 68 | 119.9 | 5.3852 | gossip |
| 34 | 162 | 118.33 | 2.1351 | bit |
| 35 | 53 | 110.8 | 7.6106 | pigeons |
| 36 | 121 | 109.04 | 2.5162 | apartment |
| 37 | 54 | 103.76 | 6.6376 | apartments |
| 38 | 91 | 97.9 | 2.9311 | beauty |
| 39 | 67 | 96.07 | 3.9488 | curtains |
| 40 | 65 | 96.02 | 4.0977 | gradually |
| 41 | 50 | 95.55 | 6.5266 | pageant |
| 42 | 94 | 93.6 | 2.7369 | simply |
| 43 | 77 | 92.15 | 3.2426 | peace |
| 44 | 70 | 91.51 | 3.5526 | hearts |
| 45 | 118 | 90.25 | 2.2108 | however |
| 46 | 77 | 86.84 | 3.062 | studio |
| 47 | 82 | 83.16 | 2.7808 | streets |
| 48 | 39 | 81.53 | 7.1681 | mahjong |
| 49 | 39 | 81.53 | 7.1681 | wouldn’t |
| 50 | 55 | 80.96 | 4.0791 | excitement |
| 51 | 62 | 80.25 | 3.515 | photo |
| 52 | 61 | 78.46 | 3.4915 | fashion |
| 53 | 43 | 75.26 | 5.309 | rumors |
| 54 | 50 | 71.59 | 3.9416 | parties |
| 55 | 34 | 71.07 | 6.9702 | cannot |
| 56 | 66 | 68.54 | 2.8396 | lane |
| 57 | 31 | 64.8 | 6.8369 | cheongsam |
| 58 | 31 | 64.8 | 6.8369 | trolley |
| 59 | 48 | 64.48 | 3.6603 | film |
| 60 | 66 | 64.11 | 2.6792 | style |
| 61 | 80 | 60.96 | 2.2046 | neither |
| 62 | 29 | 60.62 | 6.7407 | qiyaos |
| 63 | 49 | 57.4 | 3.1755 | kinds |
| 64 | 27 | 56.44 | 6.6376 | pedicab |
| 65 | 27 | 56.44 | 6.6376 | suzhou |
| 66 | 41 | 55.01 | 3.6553 | rice |
| 67 | 43 | 52.51 | 3.309 | cousin |
| 68 | 25 | 52.26 | 6.5266 | huaihai |
| 69 | 25 | 52.26 | 6.5266 | isn’t |
| 70 | 23 | 48.08 | 6.4063 | outfits |
| 71 | 43 | 47.22 | 2.987 | proper |
| 72 | 26 | 46.66 | 5.5831 | alleys |
| 73 | 54 | 45.64 | 2.3897 | future |
| 74 | 28 | 45.57 | 4.6901 | bicycle |
| 75 | 25 | 44.65 | 5.5266 | splendor |
| 76 | 21 | 43.9 | 6.275 | doesn’t |
| 77 | 45 | 43.61 | 2.6741 | buildings |
| 78 | 29 | 43.31 | 4.1557 | maid |
| 79 | 47 | 40.72 | 2.4373 | happiness |
| 80 | 36 | 40.46 | 3.0526 | western |
| 81 | 19 | 39.72 | 6.1306 | weren’t |
| 82 | 29 | 39.71 | 3.7407 | scenes |
| 83 | 38 | 38.97 | 2.8087 | fate |
| 84 | 35 | 38.78 | 3.012 | clothing |
